# Interferon-induced transmembrane proteins as biomarkers for assessing the diagnosis and severity of coronary artery disease and acute myocardial infarction

**DOI:** 10.3389/fmed.2025.1645725

**Published:** 2025-11-24

**Authors:** Qingyan Huang, Xiaoqi Zheng, Yuhong Gan, Shaobin Zhi, Junxing Zhu, Xiaoyan Tang, Mingfeng Huang, Qionghui Huang

**Affiliations:** 1 Institute of Cardiovascular Disease, Affiliated Meizhou Hospital of Shantou University Medical College, Meizhou, China; 2 Guangdong Provincial Engineering and Technological Research Center of Clinical Molecular Diagnosis and Antibody Drugs, Meizhou People’s Hospital (Huangtang Hospital), Meizhou Academy of Medical Sciences, Meizhou, China; 3 Department of Clinical Pharmacy, Meizhou People’s Hospital (Huangtang Hospital), Meizhou Academy of Medical Sciences, Meizhou, China; 4 GuangDong Engineering Technological Research Center of Molecular Diagnosis in Cardiovascular Diseases, Meizhou People’s Hospital (Huangtang Hospital), Meizhou Academy of Medical Sciences, Meizhou, China; 5 Center for Cardiovascular Diseases, Meizhou People's Hospital (Huangtang Hospital), Meizhou Academy of Medical Sciences, Meizhou, China

**Keywords:** biomarkers, coronary artery disease, acute myocardial infarction, IFITM1, IFITM2, IFITM3

## Abstract

**Backgroud:**

Biomarker discovery remains pivotal for improving coronary artery disease (CAD) and acute myocardial infraction (AMI) diagnosis. While interferon-induced transmembrane proteins (IFITM1/2/3) are established in viral defense and cancer progression, their roles in cardiovascular pathologies are undefined.

**Methods:**

A retrospective cohort study was conducted, including a discovery cohort (129 CAD patients and 20 controls), a validation cohort (40 CAD patients and 16 controls) and a third cohort of 52 patients with acute myocardial infraction (AMI). Serum IFITM1/2/3 levels were specifically quantified using enzyme linked immunosorbent assay (ELISA). Coronary stenosis severity was assessed using the Gensini score. The evaluation of diagnostic performance utilized receiver operating characteristic (ROC) curves, whereas Spearman’s rank test facilitated the analysis of correlations.

**Results:**

Compared with controls, CAD patients exhibited significantly elevated serum IFITM1/2/3 levels (*p* < 0.001). ROC analysis demonstrated exceptional diagnostic accuracy for CAD detection: IFITM1 (AUC 0.9375, sensitivity 95%, specificity 81.25%), IFITM2 (AUC 0.8984, sensitivity 90%, specificity 75%), and IFITM3 (AUC 1.000, sensitivity 97.5%, specificity 100%). IFITM levels were significantly positively correlated with Gensini scores (*p* < 0.0001), indicating a plaque burden-dependent expression pattern. AMI patients exhibited further elevation of IFITM1/2/3 compared to patients with stable CAD (*p* < 0.0001), with IFITM1 specifically upregulated in AMI with heart failure (3.07 vs. 4.64 ng/mL, *p* = 0.003).

**Conclusion:**

IFITM1/2/3 may serve as novel serum biomarkers for diagnosing CAD and AMI, as well as stratifying coronary stenosis severity, with high discriminatory capacity. Our findings position IFITMs as promising tools for precision cardiovascular risk assessment and therapeutic targeting.

## 1 Introduction

Coronary artery disease, a leading cause of mortality worldwide, has been recognized as a global public health problem (1). The underlying pathology of CAD is defined by the formation of atherosclerotic plaques, which involve complex processes such as endothelial dysfunction, lipid accumulation, inflammatory reactions, and thrombosis (2, 3). The clinical spectrum of CAD includes symptoms from stable angina and unstable angina to catastrophic consequences such as myocardial infarction (MI), heart failure (HF), or sudden cardiac death (4).

The characterization of cardiovascular disease biomarkers has become a vital field in translational medicine, serving dual roles in elucidating pathophysiological mechanisms and guiding therapeutic decision-making (5, 6). They provide profound insights into normal molecular physiology and disease activity/progression dynamics, and pharmacologists utilize them to unravel the intricacies of drug action mechanisms, including efficacy, safety, and off-target effects (7). Some biomarkers may serve as risk factors to predict the occurrence of cardiovascular events and thus become therapeutic targets (8–10).

Interferon-induced transmembrane proteins (IFITMs), conserved evolutionarily as a family of cytokines, have diverse roles in antiviral defense, immune regulation, and disease pathogenesis (11). The human IFITM family includes five functional homologs (IFITM1-5 and IFITM10), with IFITM1-3 being the most well-characterized (12). Initially identified for their potent antiviral activity through membrane compartmentalization, IFITMs have been shown by emerging studies over the past years to play roles in tumor progression, immune modulation, and the cardiovascular system (13). Their overexpression in multiple cancers promotes tumor progression via Wnt/*β*-catenin-mediated proliferation, VEGF-driven angiogenesis, and EMT-facilitated metastasis (14–16). IFITMs critically regulate immune homeostasis by coordinating innate/adaptive responses and shaping lymphocyte polarization, antibody diversification, and cytokine signaling networks. Conversely, pathological IFITM dysregulation exacerbates inflammatory cascades and promotes immune evasion (17–19). However, investigations into the roles of IFITMs in cardiovascular pathophysiology remain scarce compared to their well-characterized functions in virology and oncology. A previous study revealed overexpression of IFITM1 and IFITM3 during heart development (20). Notably, a recent genome-wide association study identified a genetic variant in IFITM2 (rs1059091) associated with an increased risk of CAD in an Indian population, providing the first genetic evidence linking the IFITM family to coronary artery disease (21). The co-occurrence of IFITM1/2/3 overexpression with Treg/neutrophil imbalance in DCM patients highlights their potential as master regulators of cardiac inflammation (22). Recently, our previous work found that IFITM1 was upregulated in arteriosclerotic plaques and contributed to arteriosclerosis progression (23).

This retrospective clinical cohort study was performed to explore the association of IFITM1/2/3 with coronary stenosis severity and to evaluate the diagnostic accuracy of circulating IFITMs for identifying CAD and AMI patients. The primary outcome was the diagnostic performance of serum IFITM1/2/3 levels in distinguishing CAD and AMI patients from healthy controls, as assessed by receiver operating characteristic (ROC) analysis. The secondary outcomes included (1) the correlation between IFITM1/2/3 levels and coronary stenosis severity quantified by Gensini score, and (2) the differential expression of IFITM1/2/3 in AMI patients with and without heart failure.

## 2 Methods

### 2.1 Study design and participants

**Figure 1:**
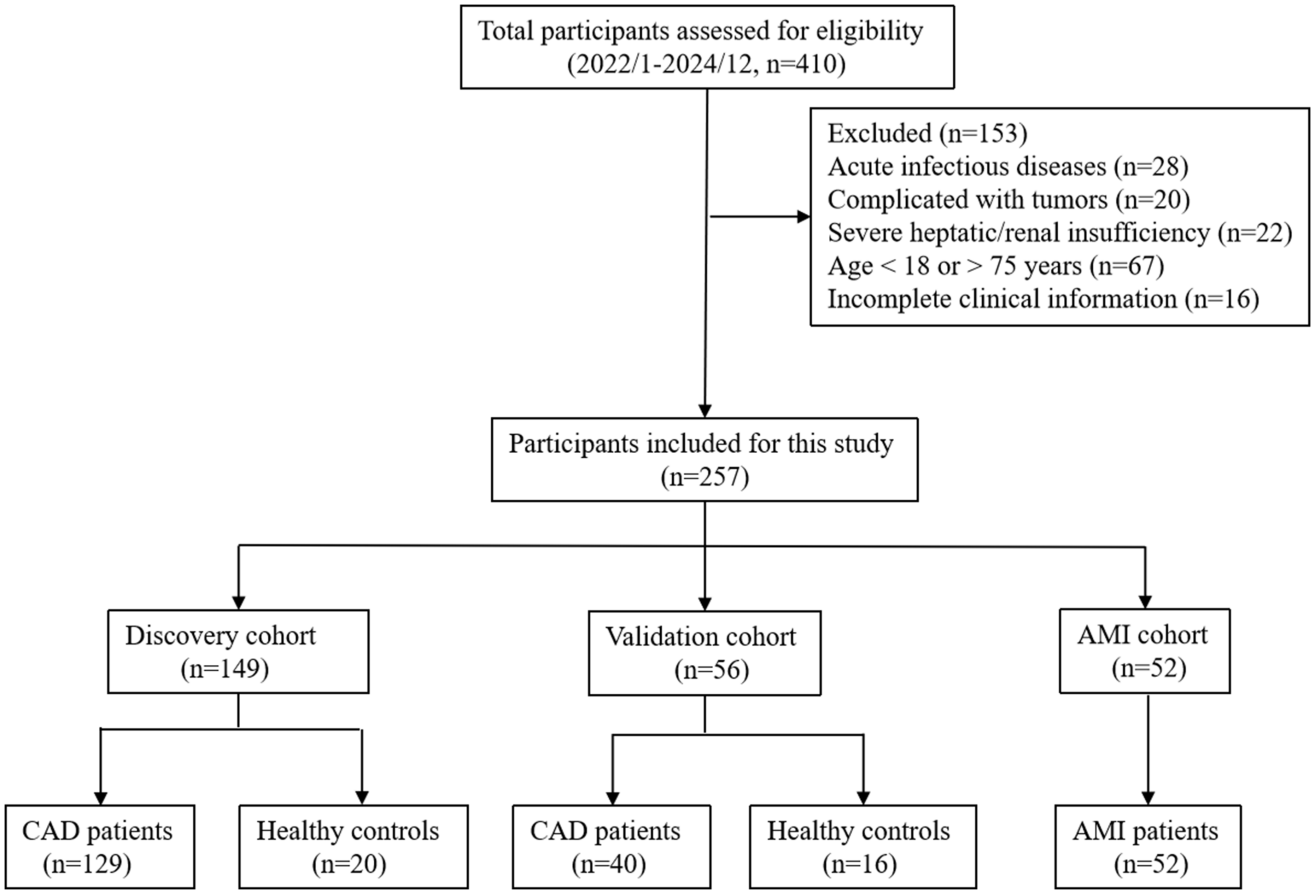
Flow chart of participants selection.

This was a single-center, retrospective cohort study. A total of 257 participants were consecutively enrolled from Meizhou People’s Hospital between January 2022 and December 2024. The study utilized a convenience sampling approach, retrospectively including all eligible patients who met the pre-specified inclusion and exclusion criteria during the study period. All individuals were divided into the CAD discovery cohort (*n* = 149), the validation cohort (*n* = 56), and the AMI cohort (*n* = 52). Exclusion conditions were: (1) acute infectious diseases; (2) complicated with any kind of tumors; (3) severe hepatic or renal insufficiency; (4) age <18 or >75 years; (5) incomplete clinical information. The control subjects with a previous history of cardiovascular diseases or systemic diseases, or those using medications affecting lipid metabolism, inflammation, or blood pressure, were excluded. The CAD subjects were diagnosed with stable coronary artery disease, defined as no acute cardiovascular events (e.g., myocardial infarction, stroke) in the prior 6 months. The specific recruitment process and grouping are presented in Figure 1. This study was approved by the Ethics Committee of Meizhou People’s Hospital (2023-C) and conducted in accordance with the Declaration of Helsinki.

### 2.2 Clinical data collection

Data on demographic factors (age, gender) and clinical parameters (patient history, pertinent diagnostic imaging studies, laboratory test outcomes) were extracted from the hospital electronic health record system. Past medical history included hypertension and diabetes. Routine laboratory tests were performed in the central clinical laboratory of our hospital. The complete blood count was analyzed using a Mindray BC-600 hematology analyzer (Mindray Bio-Medical Electronics Co., Ltd., Shenzhen, China) with its proprietary reagents. Measurements of the lipid profile, aspartate aminotransferase (AST), and alanine aminotransferase (ALT) were conducted on a Beckman Coulter AU5800 fully automated biochemical analyzer (Beckman Coulter, Inc., USA) using reagent kits from Medconn Biotechnology Co., Ltd. (Ningbo, China). All procedures followed the manufacturer’s instructions and the laboratory’s standard operating protocols.

### 2.3 Gensini score

Based on coronary angiography results, the Gensini score was used to quantify the stenosis severity in CAD. The Gensini score is calculated in two steps: first, assigning a stenosis score based on artery blockage percentage (1 for ≤25%, 2 for 26%–50%, 4 for 51%–75%, 8 for 76%–90%, 16 for 91%–99%, 32 for total occlusion); second, multiplying this score by a segment-specific coefficient. Coefficients were: 5 for the left main coronary artery; 2.5 for the proximal left anterior descending and left circumflex arteries; 1.5 for the middle left anterior descending artery; 1 for the first diagonal branch, obtuse marginal branches, and right coronary artery; and 0.5 for the second diagonal branch and left circumflex posterolateral branch (24). Scores were independently assigned by two senior cardiologists. Patients were stratified into Gensini-low (*n* = 63) and Gensini-high (*n* = 66) groups according to median Gensini score.

### 2.4 Measurement of IFITM1/2/3 serum concentrations

Serum levels of IFITM1/2/3 were measured using ELISA kits (EH9298, EH9300, EH1111; FineTest) according to the manufacturer’s protocols. Serum samples were diluted 1:2 with dilution buffer prior to analysis. Absorbance was measured at 450 nm. The concentration in samples was calculated using CurveExpert1.4 software (Danie Hyams).

### 2.5 Statistical analysis

With R (version 4.2.2) and GraphPad Prism (version 9.0), statistical analyses were performed. Data normality was assessed using the Shapiro–Wilk test. Normally distributed variables were described as mean ± SD and compared using Student’s *t*-test or one-way ANOVA. Non-normally distributed variables were expressed as median (interquartile range) and compared using the Wilcoxon rank-sum test or Kruskal–Wallis *H* test. Categorical variables, expressed as percentages, were assessed by the Chi-square test. The association between IFITM levels and the Gensini score was analyzed using Spearman’s correlation. The diagnostic performance of serum IFITMs was evaluated by ROC curve analysis, with the area under the curve (AUC) and 95% confidence interval reported. The optimal cutoff value for each biomarker was determined by maximizing Youden’s index. To ensure robustness, the diagnostic models and cut-offs established in the discovery cohort were validated in an independent validation cohort. A *p*-value < 0.05 was defined as statistically significant.

## 3 Results

### 3.1 Baseline characteristics of subjects

Baseline characteristics of discovery and validation cohorts are tabulated in Table 1. In the discovery cohort, CAD patients had higher neutrophils, lymphocytes, and Apolipoprotein B levels compared with controls (*p* < 0.05). Other parameters showed no significant group differences. In the validation cohort, white blood cell count, neutrophils, lymphocytes, and triglycerides differed significantly between groups (*p* < 0.05).

**Table 1:** The demographic and clinical features of discovery and validation cohort.

Variables	Discovery cohort		Validation cohort			
	Control (*n* = 20)	CAD (*n* = 129)	*p*-value	Control (*n* = 16)	CAD (*n* = 40)	*p*-value
Demographics						
Gender			0.68			0.577
Female (*n*, %)	5 (25.00%)	43 (33.33%)		8 (50%)	15 (37.5%)	
Male (*n*, %)	15 (75.00%)	86 (66.67%)		8 (50%)	25 (62.5%)	
Age (years)	61.50 (56.00, 66.00)	66.00 (64.00, 69.00)	0.5	58.50 (56.75, 60.00)	64.50 (62.00–67.25)	0.091
Medical history						
Diabetes (*n*, %)	/	41 (32%)		/	9 (23%)	/
Hypertension (*n*, %)	/	89 (69%)		/	27 (68%)	/
Laboratory examination						
White blood cells (× 10^9^/L)	6.00 (5.58, 7.38)	7.10 (5.60, 8.70)	0.066	6.10 (5.33, 6.30)	7.50 (5.80, 8.65)	0.03
Neutrophils (× 10^9^/L)	3.26 (2.52, 4.74)	4.66 (3.67, 5.94)	0.001	3.32 (2.85, 3.66)	4.72 (4.04, 6.09)	<0.001
Lymphocytes (× 10^9^/L)	2.13 (1.76, 2.45)	1.63 (1.25, 2.11)	0.005	2.17 (1.61, 2.38)	1.56 (1.35, 2.07)	0.033
Monocytes (× 10^9^/L)	0.42 (0.30, 0.53)	0.44 (0.36, 0.57)	0.304	0.37 (0.32, 0.42)	0.43 (0.34, 0.60)	0.102
Red blood cells (× 10^10^/L)	4.82 (4.55, 5.09)	4.57 (4.16, 4.94)	0.055	4.77 (4.31, 5.00)	4.51 (4.12, 4.84)	0.185
Platelets (× 10^9^/L)	206.00 (193.00, 251.00)	224.00 (182.00, 262.00)	0.804	224.50 (212.0, 234.75)	215.00 (203.00, 255.00)	0.935
Triglycerides (mmol/L)	1.22 (1.06, 1.44)	1.42 (1.03, 1.99)	0.074	1.06 (0.92, 1.14)	1.72 (1.17, 2.25)	0.006
Total cholesterol (mmol/L)	4.27 (3.76, 4.89)	4.45 (3.86, 5.34)	0.331	4.75 (4.54, 5.01)	4.68 (4.11, 5.39)	0.814
High-density lipoprotein (mmol/L)	1.46 (1.33, 1.56)	1.36 (1.12, 1.67)	0.3	1.42 (1.34, 1.61)	1.25 (1.05, 1.45)	0.118
Low-density lipoprotein (mmol/L)	2.62 (2.04, 2.87)	2.66 (2.28, 3.40)	0.13	2.76 (2.54, 2.95)	2.90 (2.55, 3.29)	0.245
Apolipoprotein A1 (g/L)	1.16 (1.08, 1.31)	1.21 (1.03, 1.41)	0.902	1.28 (1.23, 1.36)	1.10 (0.95, 1.32)	0.094
Apolipoprotein B (g/L)	0.71 (0.62, 0.76)	0.84 (0.72, 1.04)	0.006	0.76 (0.72, 0.86)	0.88 (0.75, 1.12)	0.053
Markers						
IFITM1 (ng/mL)	1.32 (1.11, 1.44)	2.63 (2.06, 3.21)	<0.001	1.42 (1.28, 1.57)	2.32 (2.01, 3.54)	<0.001
IFITM2 (ng/mL)	4.20 (3.55, 4.92)	9.03 (7.09, 12.13)	<0.001	4.11 (3.88, 5.60)	9.34 (7.33, 10.80)	<0.001
IFITM3 (pg/mL)	2112.02 (1865.59, 2249.60)	3419.85 (3076.43, 4367.26)	<0.001	2084.72 (1910.07, 2262.84)	3445.12 (3038.17, 3939.96)	<0.001

Data are presented as mean ± SD, median (IQR), or *n* (%) as appropriate; group comparisons were performed using Student’s *t*-test, Wilcoxon rank-sum test, or Chi-square test based on data distribution and variable type.

### 3.2 Analysis of serum IFITM1/2/3 levels for CAD diagnosis

**Figure 2:**
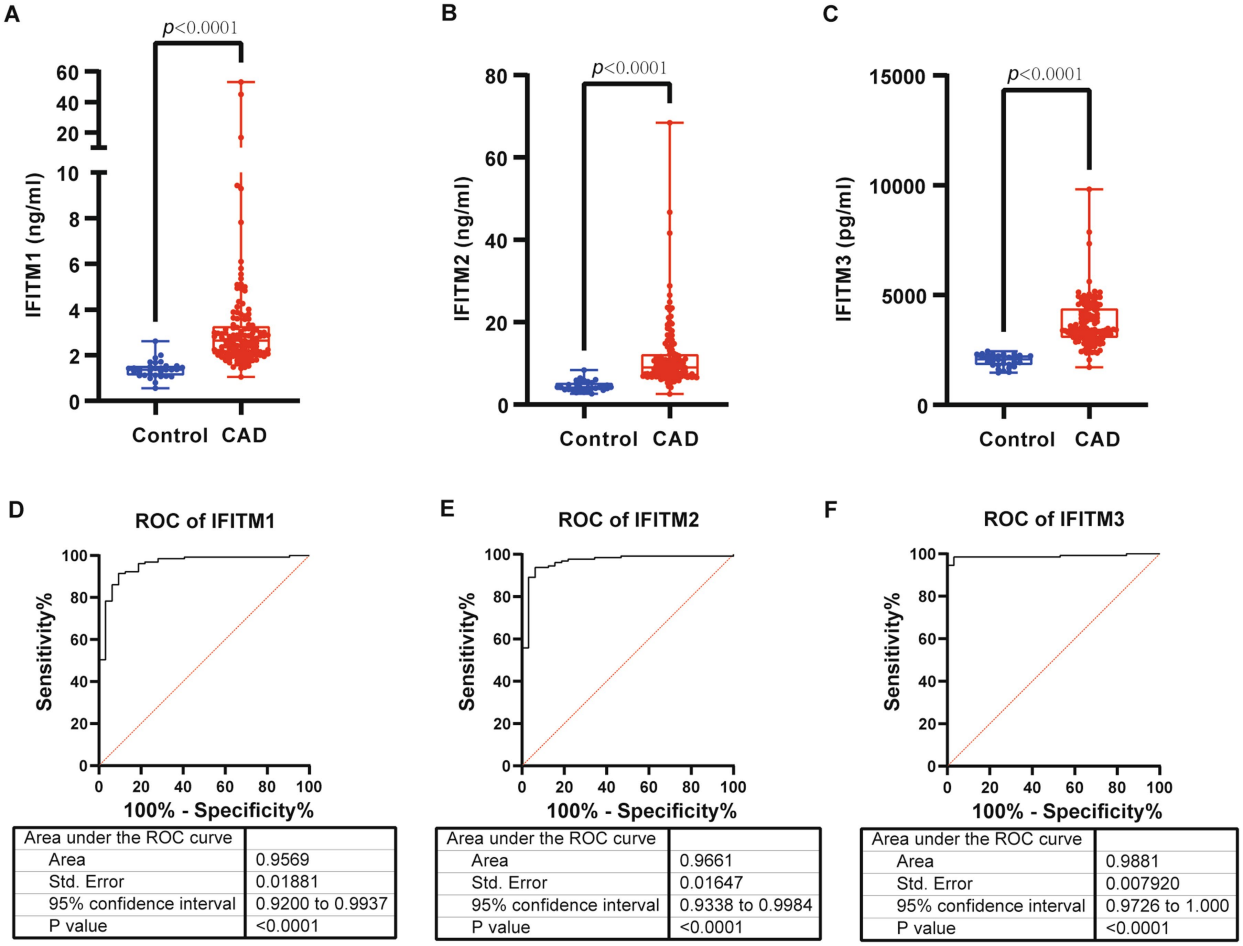
Serum levels and diagnostic performance of IFITM1/2/3 in control and CAD group of discovery cohort. **(A–C)** Serum IFITM1/2/3 in control and CAD subjects; **(D–F)** ROC curves of IFITM1/2/3 for determination of CAD. Statistical significance was assessed using Wilcoxon rank-sum test.

**Figure 3:**
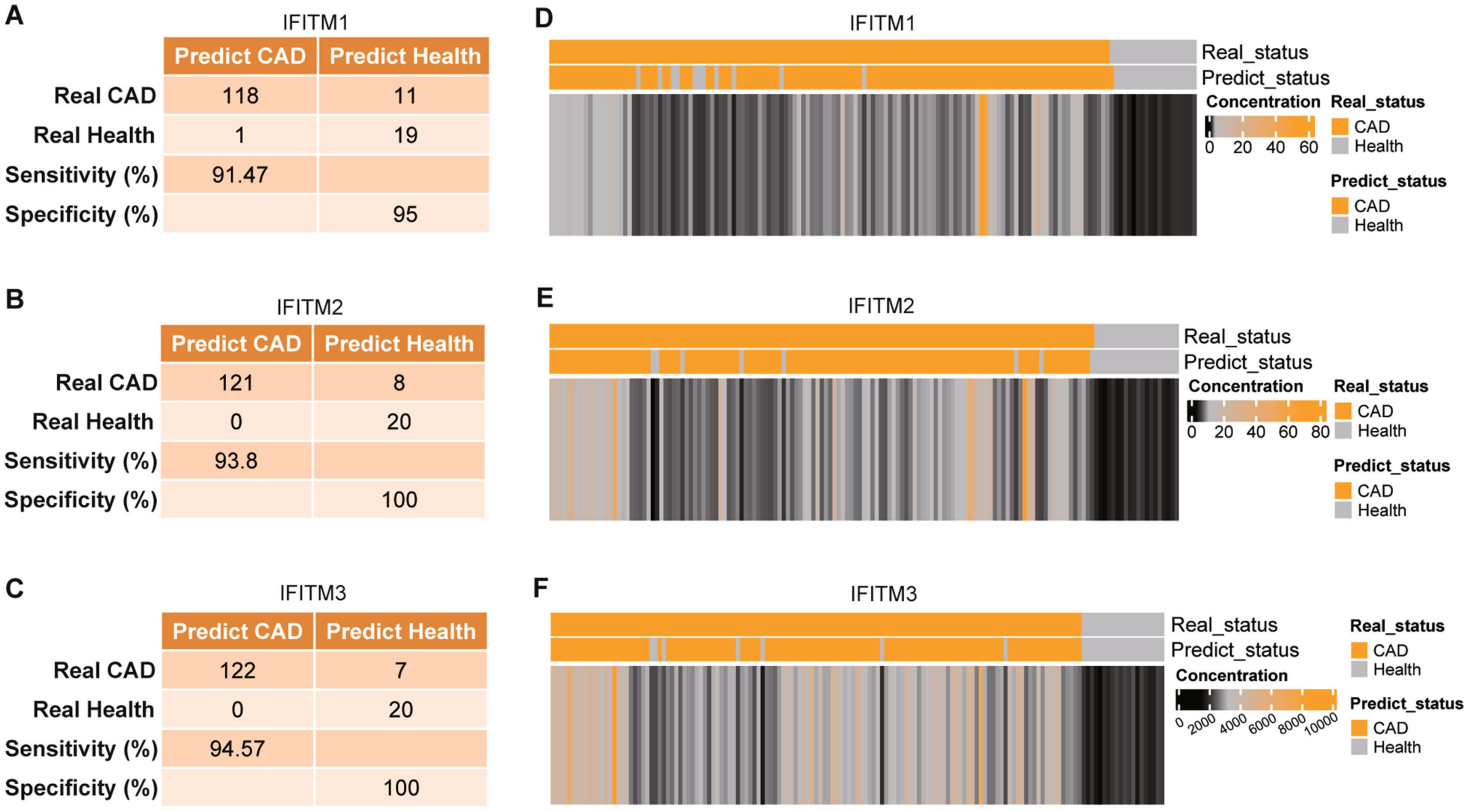
Diagnostic ability of IFITM1/2/3 in discovery cohort. **(A–C)** Confusion tables of the binary results of IFITM1/2/3; **(D–F)** Supervised hierarchical clustering of IFITM1/2/3 between health control and CAD.

Serum levels of IFITM1/2/3 were analyzed in the discovery cohort. CAD patients exhibited significantly higher serum levels of IFITM1/2/3 compared with healthy controls (Figures 2A–C). ROC curves confirmed diagnostic efficacy in three proteins, with area under the curve (AUC) values of 0.9569 (95% CI: 0.9200–0.9937; cutoff >1.737 ng/mL, sensitivity 91.47%, specificity 95%) for IFITM1, 0.9661 (95% CI: 0.9338–0.9984; cutoff >6.092 ng/mL, sensitivity 93.8%, specificity 100%) for IFITM2, and 0.9881 (95% CI: 0.9726–1.000; cutoff >2,452 pg./mL, sensitivity 94.57%, specificity 100%) for IFITM3 (Figures 2D–F, 3A–C). Notably, IFITM proteins had optimal diagnostic thresholds determined by Youden’s index, which demonstrated robust discriminatory power in distinguishing CAD patients from healthy controls (Figures 3D–F).

### 3.3 Diagnostic discrimination of CAD patients by IFITM1/2/3 from healthy controls

For validation of IFITM proteins’ diagnostic potential, a validation cohort (16 healthy controls, 40 CAD patients) was prospectively analyzed. Consistent with the discovery cohort results, serum levels of IFITM1/2/3 were significantly higher in CAD patients compared with controls (Supplementary Figure S1A–C). ROC analysis confirmed strong diagnostic discrimination, with IFITM1 showing an AUC of 0.9375 (95% CI: 0.8632–1.000; sensitivity 95%, specificity 81.25%), IFITM2 an AUC of 0.8984 (95% CI: 0.8044–1.000; sensitivity 90%, specificity 75%), and IFITM3 a perfect AUC of 1.000 (95% CI: 1.000–1.000; sensitivity 97.5%, specificity 100%) (Supplementary Figures S1D–F, S2A–C). Furthermore, when applying the thresholds from discovery cohort to the validation cohort, all three proteins retained robust classification performance in distinguishing CAD status (Supplementary Figure S2D–F).

### 3.4 Association of IFITM1/2/3 levels with coronary stenosis severity

**Figure 4:**
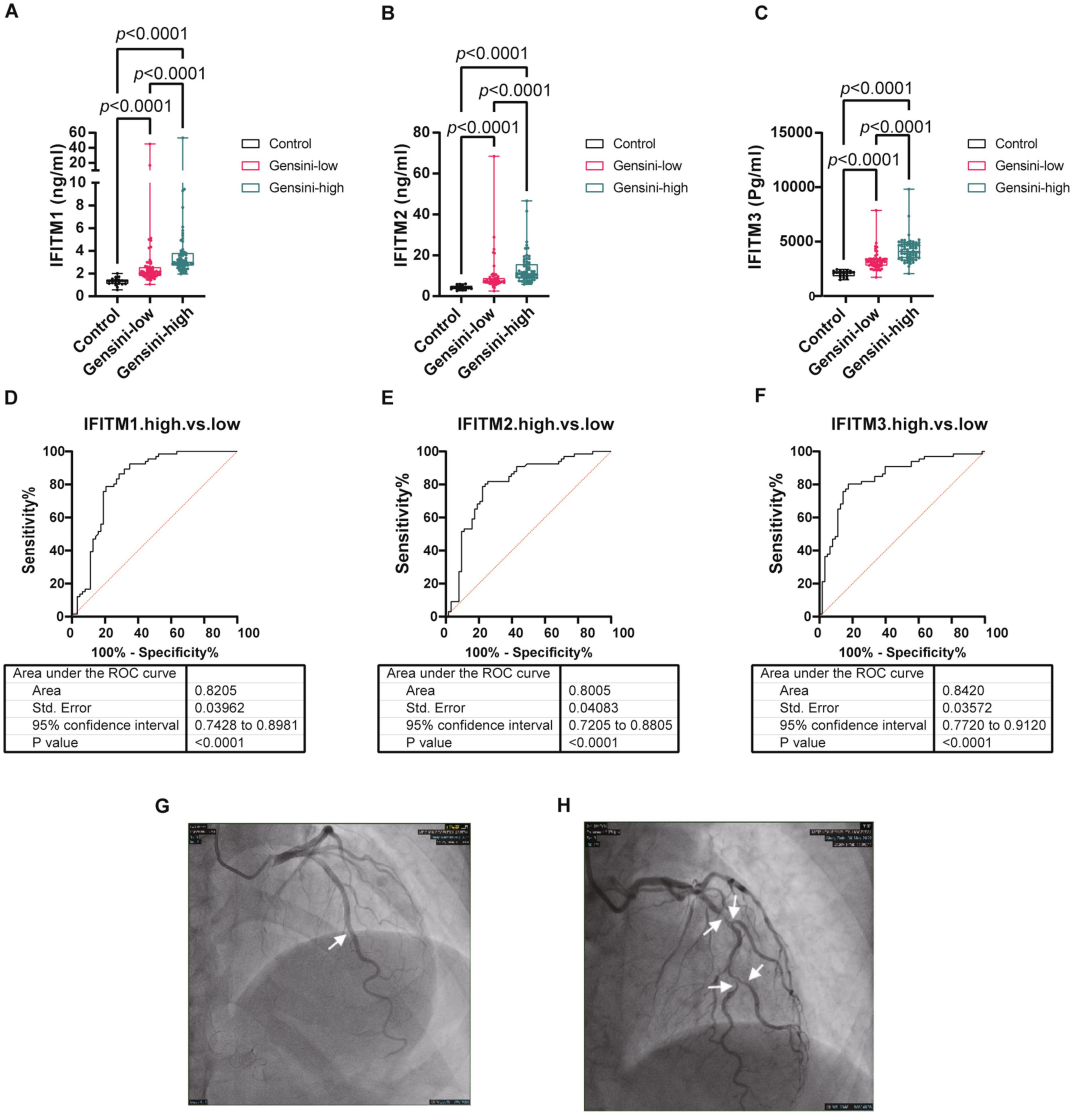
Levels of serum IFITM1/2/3 among different coronary severity groups of discovery cohort. **(A–C)** Compared IFITM1/2/3 levels in control, Gensini-low and Gensini-high groups; **(D–F)** ROC curves of IFITM1/2/3 for determination of different coronary severity CAD patients; **(G–H)** Representative coronary angiography images. Statistical significance was assessed using Kruskal–Wallis *H* test.

**Figure 5:**
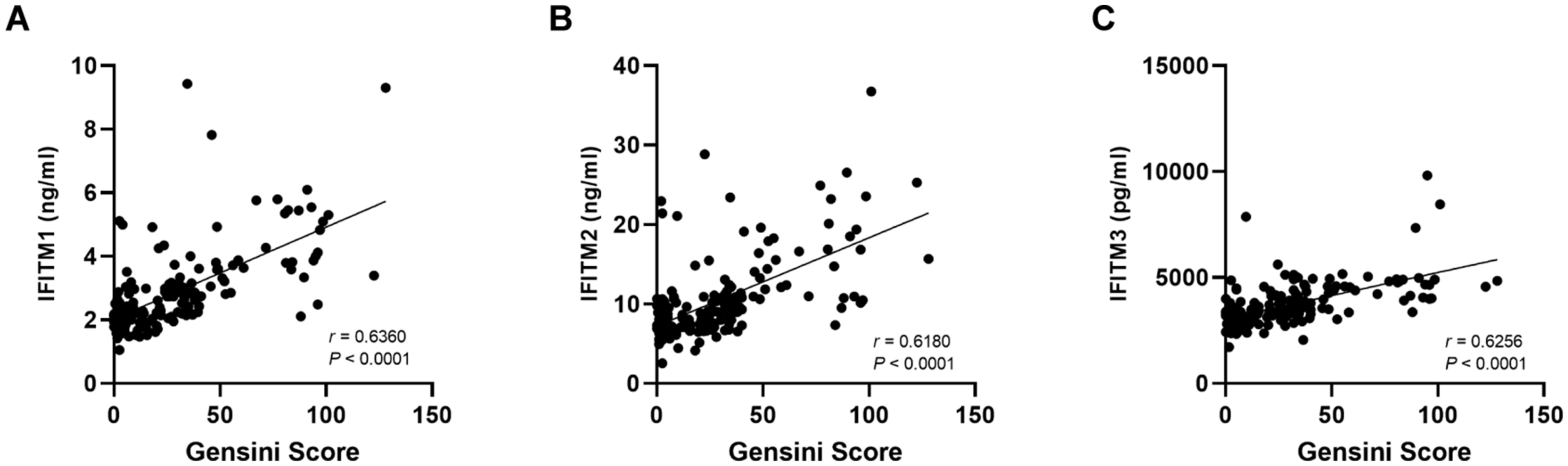
Correlation of serum IFITM1/2/3 with Gensini score. **(A–C)** The correlation results of IFITMs between Gensini score.

For further investigation of IFITM protein levels and CAD severity association, 129 discovery cohort CAD patients were stratified by median Gensini score into low/high-stenosis severity groups. Figures 4G–H display representative coronary angiography images depicting varying stenosis degrees in the two subgroups. Clinical characteristics of the two groups were shown in Table 2. Serum IFITM concentrations showed a gradual increase from the Gensini-low to Gensini-high group (Figures 4A–C). These results suggest a positive correlation between IFITM protein levels and coronary artery stenosis severity. This gradient pattern showed a significant correlation with angiographic severity scores (Figures 5A–C). Furthermore, ROC curve analysis showed that IFITMs exhibited strong discriminatory ability in distinguishing low vs. high stenosis severity, with an AUC of 0.8 (Figures 4D–H).

**Table 2:** Demographic characteristics between GS-low and GS-high group.

Variables	Gensini-low (*n* = 63)	Gensini-high (*n* = 66)	*p*-value
Demographics			
Gender			0.575
Female (*n*, %)	19 (30.16%)	24 (36.36%)	
Male (*n*, %)	44 (69.84%)	42 (63.64%)	
Age (year)	66.00 (64.50–67.50)	68.00 (64.00–70.75)	0.056
Height (cm)	162.50 (155.75–167.75)	162.00 (155.00–166.00)	0.518
Weight (kg)	61.50 (57.00–71.00)	64.00 (59.00–70.00)	0.461
Gensini Score	5.50 (2.50–14.50)	37.00 (32.00–55.75)	<0.001
Medical history			
Diabetes (*n*, %)	17 (27%)	24 (36%)	0.255
Hypertension (*n*, %)	46 (73%)	43 (65%)	0.336
Laboratory examination			
White blood cells (× 10^9^/L)	6.90 (5.50–8.70)	7.25 (6.03–8.50)	0.472
Neutrophils (× 10^9^/L)	4.55 (3.54–6.46)	4.68 (3.81–5.64)	0.759
Lymphocytes (× 10^9^/L)	1.43 (1.19–1.83)	1.81 (1.36–2.18)	0.025
Monocytes (× 10^9^/L)	0.44 (0.36–0.57)	0.48 (0.36–0.57)	0.561
Red blood cells (× 10^10^/L)	4.58 (4.12–4.96)	4.56 (4.21–4.90)	0.845
Platelets (× 10^9^/L)	215.00 (171.50–253.50)	232.00 (196.75–270.25)	0.14
Fibrinogen (g/L)	3.15 (2.80–3.65)	3.20 (2.90–3.93)	0.217
D Dimer (mg/L)	0.35 (0.27–0.62)	0.32 (0.25–0.51)	0.47
C reactive protein (mg/L)	2.14 (0.92–3.10)	1.47 (0.91–5.28)	0.986
Creatinine (μmol/L)	88.00 (71.90–101.00)	80.45 (63.65–96.85)	0.289
Procalcitonin (ng/mL)	0.05 (0.05–0.05)	0.05 (0.05–0.05)	0.584
Alanine aminotransferase (U/L)	21.00 (15.00–28.50)	19.50 (15.00–25.00)	0.524
Aspartate aminotransferase (U/L)	21.00 (17.00–25.50)	20.50 (17.25–25.00)	0.85
Triglycerides (mmol/L)	1.51 (0.97–1.90)	1.38 (1.06–2.05)	0.923
Total cholesterol (mmol/L)	4.47 (3.80–5.33)	4.43 (3.95–5.33)	0.796
High-density lipoprotein (mmol/L)	1.49 (1.11–1.79)	1.33 (1.19–1.53)	0.1
Low-density lipoprotein (mmol/L)	2.63 (2.25–3.37)	2.66 (2.40–3.55)	0.276
Apolipoprotein A1 (g/L)	1.21 (1.05–1.44)	1.21 (1.03–1.37)	0.505
Apolipoprotein B (g/L)	0.82 (0.71–1.05)	0.90 (0.73–1.04)	0.394
Homocysteine (μmol/L)	10.75 (9.07–12.97)	10.95 (8.90–12.94)	0.823
Glycated hemoglobin (%)	6.30 (5.88–6.80)	6.20 (5.80–7.05)	0.715
Markers			
IFITM1 (ng/mL)	2.11 (1.82–2.53)	2.98 (2.73–3.77)	<0.001
IFITM2 (ng/mL)	7.23 (6.63–8.76)	10.93 (9.06–15.54)	<0.001
IFITM3 (pg/mL)	3149.47 (2808.80–3397.46)	4088.63 (3515.54–4746.90)	<0.001

Data are presented as mean ± SD, median (IQR), or *n* (%) as appropriate; group comparisons were performed using Student’s *t*-test, Wilcoxon rank-sum test, or Chi-square test based on data distribution and variable type.

### 3.5 Comparison of serum IFITM1/2/3 levels in CAD and AMI groups

**Figure 6:**
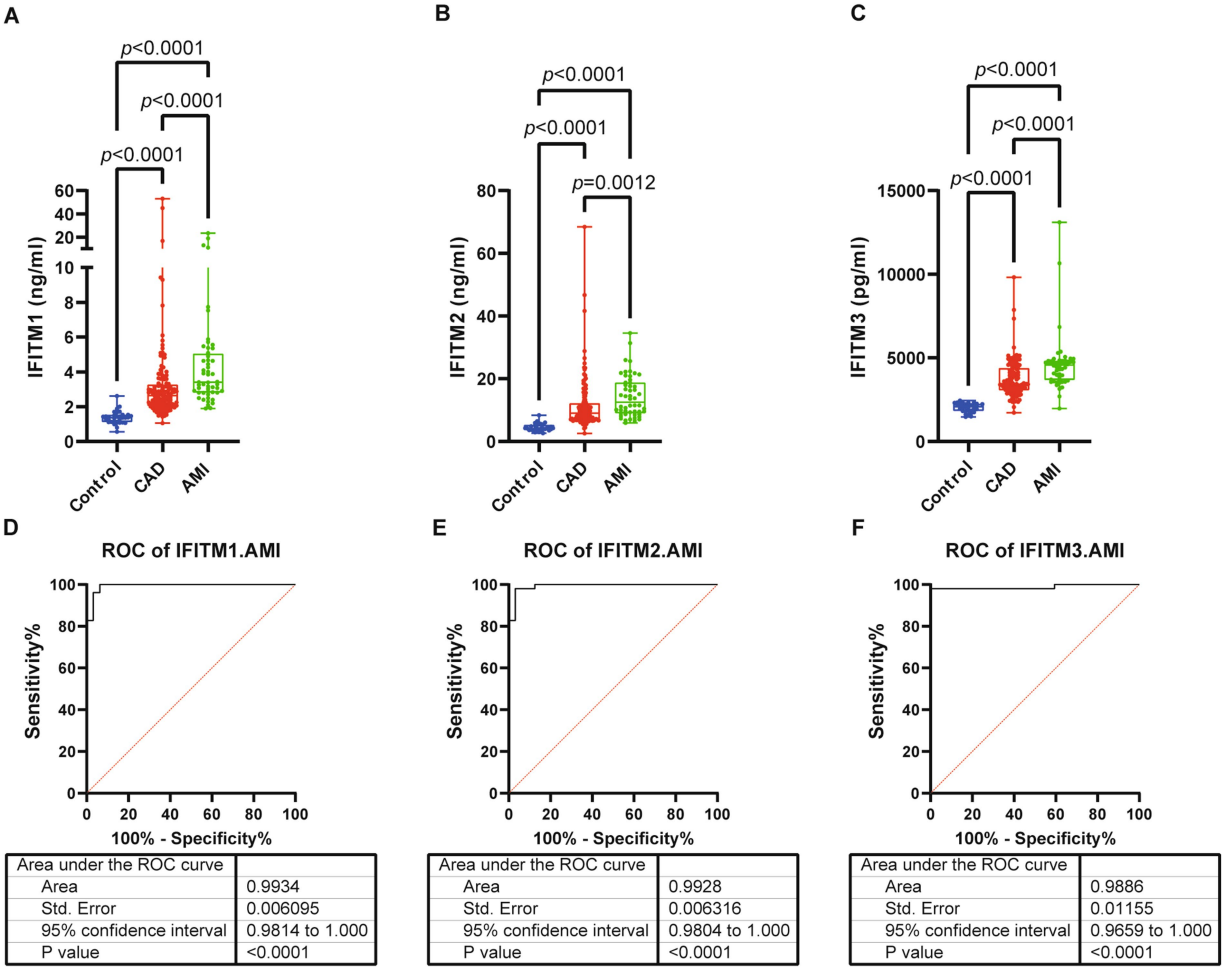
Serum levels and diagnostic performance of IFITM1/2/3 in control, CAD and AMI subjects. **(A–C)** Serum IFITM1/2/3 among three groups; **(D–F)** ROC curves of IFITM1/2/3 for determination of AMI from controls. Statistical significance was assessed using using Kruskal–Wallis H test.

**Figure 7:**
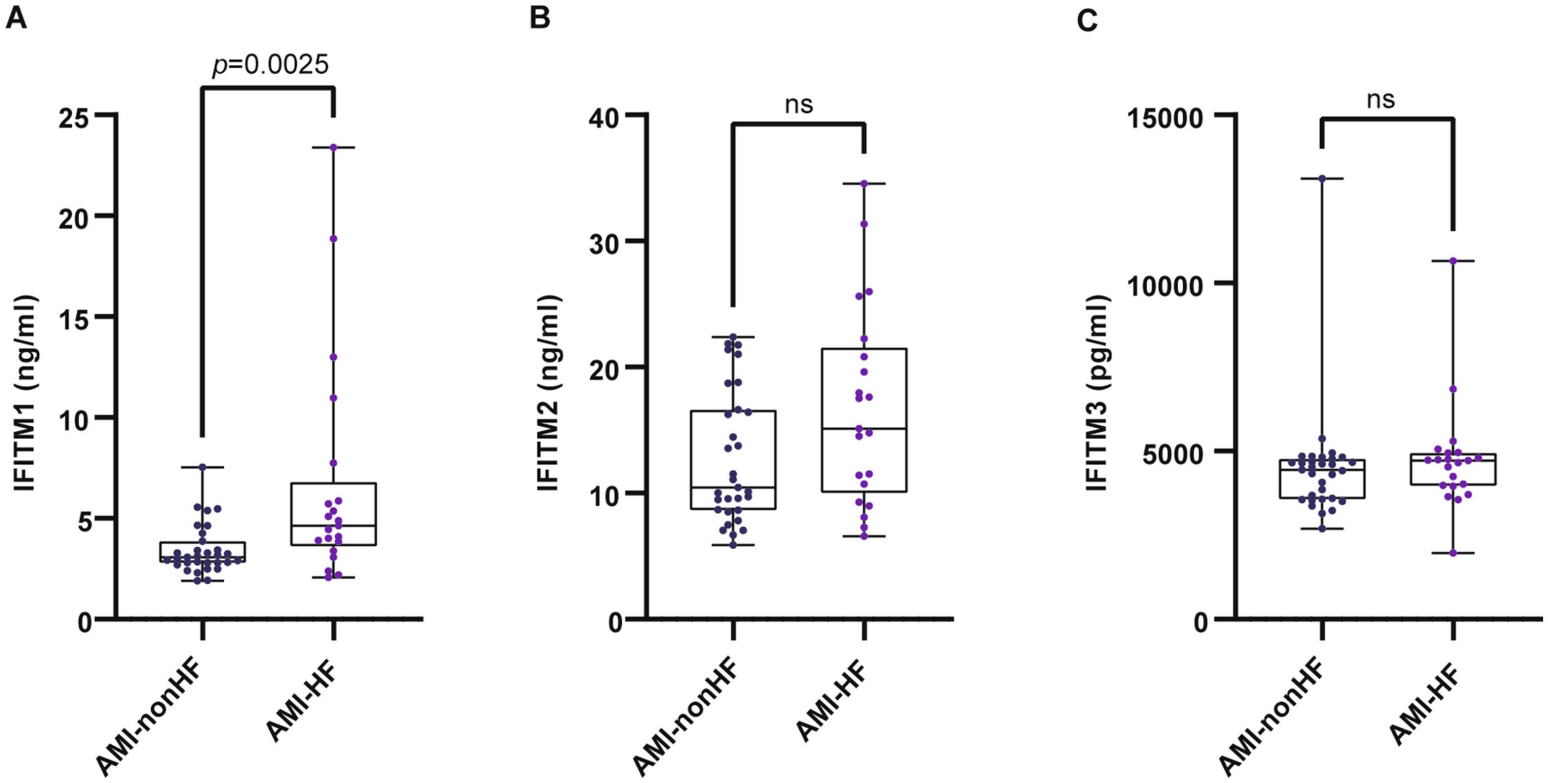
Serum levels of IFITM1/2/3 in AMI-nonHF and AMI-HF groups. **(A–C)** Compared IFITMs in AMI-nonHF and AMI-HF groups. Statistical significance was assessed using Wilcoxon rank-sum test.

Subsequent analyses compared serum IFITM1/2/3 concentrations among CAD, AMI and control groups. Baseline clinical characteristics were detailed in Table 3. Comparative analysis showed significantly higher IFITM1/2/3 levels in AMI patients compared with CAD and control groups (Figures 6A–C). ROC curves indicated high diagnostic value of serum IFITM1/2/3 for distinguishing AMI from controls, with AUC values of 0.9934, 0.9928 and 0.9886, respectively (Figures 6D–F). Further stratification of AMI patients into heart failure (AMI-HF) and non-heart failure (AMI-nonHF) subgroups (clinical characteristics detailed in Supplementary Table S1) revealed distinct expression patterns. Notably, IFITM1 levels were significantly higher in AMI-HF patients (2.41 ± 0.38 ng/mL vs. 1.79 ± 0.29 ng/mL, *p* = 0.007), while IFITM2 and IFITM3 showed no significant expression differences between subgroups (Figures 7A–C). This differential expression profile suggests a potential pathophysiological role for IFITM1 in post-infarction ventricular remodeling and heart failure development, whereas IFITM2/3 appear more broadly associated with acute coronary syndrome pathogenesis. These findings underscore the proteins’ superior discriminatory capacity in acute coronary syndromes and suggest a distinct role for IFITM1 in heart failure progression after acute myocardial infarction.

**Table 3:** The demographic and clinical features of participants among three groups.

Variables	Control (*n* = 20)	CAD (*n* = 129)	AMI (*n* = 52)	*p*-value
Demographics				
Gender				0.24
Female (*n*, %)	5 (25.00%)	43 (33.33%)	11 (21.15%)	
Male (*n*, %)	15 (75.00%)	86 (66.67%)	41 (78.85%)	
Age (year)	61.50 (56.00–66.00)	66.00 (64.00–69.00)	66.50 (64.00–69.25)	0.051
Gensini score	/	24.00 (6.00–37.00)	65.00 (39.25–81.25)	<0.001
Killp				/
I	/	/	26 (50.00%)	
II	/	/	18 (34.62%)	
III	/	/	6 (11.54%)	
IV	/	/	2 (3.85%)	
NA	20 (100.00%)	129 (100.00%)	/	
Medical history				
Diabetes (*n*, %)	/	41 (32%)	16 (37%)	0.6
Hypertension (*n*, %)	/	89 (69%)	40 (82%)	0.13
Laboratory examination				
White blood cells (× 10^9^/L)	6.00 (5.58–7.38)	7.10 (5.60–8.70)	8.95 (7.35–10.65)	<0.001
Neutrophils (× 10^9^/L)	3.26 (2.52–4.74)	4.66 (3.67–5.94)	7.00 (5.17–8.86)	<0.001
Lymphocytes (× 10^9^/L)	2.13 (1.76–2.45)	1.63 (1.25–2.11)	1.29 (0.88–1.73)	<0.001
Monocytes (× 10^9^/L)	0.42 (0.30–0.53)	0.44 (0.36–0.57)	0.51 (0.38–0.66)	0.133
Red blood cells (× 10^10^/L)	4.82 (4.55–5.09)	4.57 (4.16–4.94)	4.46 (4.20–4.80)	0.07
Platelets (× 10^9^/L)	206.00 (193.00–251.00)	224.00 (182.00–262.00)	205.00 (172.25–250.25)	0.263
Troponin (ng/mL)	/	/	6.27 (0.70–31.56)	/
B type natriuretic peptide (pg/mL)	/	35.50 (14.60–80.70)	209.50 (66.30–687.98)	<0.001
C reactive protein (mg/L)	/	1.76 (0.90–3.82)	18.13 (4.61–35.13)	<0.001
Creatinine (μmol/L)	/	82.30 (67.90–99.90)	83.00 (72.30–94.63)	0.814
Alanine aminotransferase (U/L)	/	20.00 (15.00–26.25)	29.00 (20.50–52.50)	<0.001
Aspartate aminotransferase (U/L)	21.50 (17.00–24.75)	21.00 (17.00–25.00)	45.00 (27.50–104.00)	<0.001
Creatine kinase (U/L)	/	87.00 (65.00–115.00)	248.50 (109.75–814.00)	<0.001
Creatine kinase MB Isoenzyme (U/L)	/	15.10 (12.00–19.40)	30.85 (20.88–100.10)	<0.001
Triglycerides (mmol/L)	1.22 (1.06–1.44)	1.42 (1.03–1.99)	1.58 (1.10–2.08)	0.137
Total Cholesterol (mmol/L)	4.27 (3.76–4.89)	4.45 (3.86–5.34)	4.28 (3.74–5.15)	0.486
High-density lipoprotein (mmol/L)	1.46 (1.33–1.56)	1.36 (1.12–1.67)	1.20 (1.02–1.40)	0.001
Low-density lipoprotein (mmol/L)	2.62 (2.04–2.87)	2.66 (2.28–3.40)	2.76 (2.36–3.26)	0.226
Apolipoprotein A1 (g/L)	1.16 (1.08–1.31)	1.21 (1.03–1.41)	1.01 (0.92–1.26)	0.002
Apolipoprotein B (g/L)	0.71 (0.62–0.76)	0.84 (0.72–1.04)	0.89 (0.74–1.04)	0.013

Data are presented as mean ± SD, median (IQR), or *n* (%) as appropriate; group comparisons were performed using Student’s *t*-test, Wilcoxon rank-sum test, or Chi-square test based on data distribution and variable type.

## 4 Discussion

Coronary artery disease persists as a global health challenge with pressing demands for innovative biomarkers to refine diagnostic precision and therapeutic monitoring. Our study showed that serum IFITM1/2/3 levels were significantly elevated in both stable CAD and AMI patient cohorts and positively correlated with stenosis severity. ROC analysis indicated strong diagnostic performance of all three IFITMs in distinguishing CAD or AMI from controls, with IFITM3 showing superior discriminative ability for CAD. Notably, elevated IFITM1 levels in post-AMI heart failure patients suggested its potential role in post-AMI cardiac decompensation mechanisms.

IFITM1, IFITM2, and IFITM3, the most structurally conserved members of the interferon-induced transmembrane (IFITM) family, have been extensively studied in antiviral defense and oncology (13, 25, 26). IFITM protein levels correlated with coronary stenosis severity (Gensini score) indicated their potential role in CAD pathophysiology. The progressive increase in IFITM levels with increasing stenosis severity suggested that these proteins may be involved in the pathological processes underlying atherosclerosis, such as endothelial dysfunction, inflammation, and thrombosis. Interferon (IFN)-dependent and IFN-independent mechanisms control the expression of IFITM genes in inflammatory conditions. In canonical IFN signaling, the JAK/STAT pathway is activated. This process induces IFITM genes and other interferon-stimulated genes (ISGs) (27). Modulation of IFITM expression extends beyond IFN-mediated mechanisms to include other cytokines (e.g., IL-6, TNF-α, angiotensin II) and molecules like LPS (28–30). This dual regulatory architecture enables context-specific IFITM induction across diverse inflammatory environments, potentially linking their functions to broader pathophysiological processes such as vascular inflammation or cytokine storm responses. A recent integrative multi-omics study further underscores the importance of STAT1-driven transcriptional programs in macrophages within atherosclerotic plaques, which aligns with the potential upstream regulation of IFITMs in cardiovascular inflammation (31). Building on the association with CAD and atherosclerosis, our group investigated a specific functional role. We demonstrated that IFITM1 contributes directly to atherosclerosis by regulating the phenotypic switch of vascular smooth muscle cells (VSMCs). Mechanistically, IFITM1 promotes VSMC proliferation, migration, and macrophage-like transdifferentiation through activation of the c-Src/MAPK/GATA2/E2F2 signaling pathway, thereby driving atherosclerotic plaque development (23).

In addition to their diagnostic potential, our findings also suggest a role for IFITM proteins in the pathogenesis of AMI. Significantly higher IFITM1/2/3 levels in AMI patients compared with CAD and control groups suggest their involvement in acute myocardial infarction responses. AMI, from coronary artery obstruction, causes myocardial ischemia-hypoxia, cardiac dysfunction, and detrimental effects: myocardial damage, heart failure, ventricular remodeling, reduced systolic function (32). Cellular death is a hallmark of AMI pathology. Damaged cells release their intracellular contents, including damage-associated molecular patterns (DAMPs), into the extracellular space. The immune system detects these DAMPs, initiating an inflammatory response (33, 34). Sustained and dysregulated inflammatory responses may exacerbate myocardial tissue damage and drive maladaptive cardiac remodeling. Thus, elevated inflammation in AMI may explain the higher IFITM levels in AMI patients compared with CAD patients. This notion is supported by a single-cell transcriptomic study in a murine MI model, which identified a distinct subpopulation of cardiac-infiltrating neutrophils characterized by high expression of Ifitm1, suggesting a specific role for IFITM1 in the local immune response following ischemic injury (35).

Another notable finding is that IFITM1 is selectively upregulated in post-AMI heart failure and negatively correlated with Ejection Fraction (EF) or Fractional Shortening (FS) (Supplementary Figure S3), which warrants attention. Preliminary studies suggested that IFITM may play a role in modulating cardiac function. Bin et al. (36) showed that IFITM1, a key immune-related gene in dilated cardiomyopathy (DCM), was significantly upregulated in T-cell-related subpathways, suggesting its potential role in disease progression via T-cell-mediated immune dysfunction. Furthermore, Xu et al.’s study (22) identified IFITM1, IFITM2, and IFITM3 as immune-related differentially expressed genes in DCM, implying that their aberrant expression may contribute to myocardial dysfunction through dysregulated immune-inflammatory pathways. Additionally, Xiong et al. (37) elucidated the specific mechanism of action of IFITM3, confirming that it exacerbates myocardial injury associated with myocarditis by activating the JAK2/STAT3 signaling pathway. Based on current evidence, we speculate that IFITMs may regulate myocardial function through immune-inflammatory signaling pathways, thereby contributing to post-infarction heart failure.

Collectively, our findings indicate that IFITM proteins could serve as novel, efficient, and promising diagnostic biomarkers for CAD and AMI. Unlike conventional imaging (invasive or radiation-based), IFITM testing offers non-invasive blood-based diagnosis with superior accuracy and operational efficiency. Compared with established serum biomarkers like C-reactive protein (CRP) and lipid markers, IFITM proteins show unique utility in stratifying disease severity in CAD.

Our study has several limitations. First, its single-center design and the recruitment of patients from a tertiary hospital introduce a substantial risk of referral bias. Our cohort likely represents a population with more severe or complex disease, which may limit the generalizability of our findings to community-based or primary care settings with milder cases. Second, the retrospective design, while suitable for this initial exploratory study, inherently restricts causal inference between IFITM levels and CAD/AMI. Third, despite our efforts to control for key clinical variables through multivariable analysis, the possibility of residual or unmeasured confounding persists. Factors such as detailed medication history (e.g., statins), lifestyle factors, and other unassessed inflammatory markers could potentially influence both IFITM expression and disease status. Fourth, as an initial study, the sample size was determined by data availability. Although a post-hoc analysis indicated high statistical power for our primary findings, the sample size, particularly of the validation cohort, remains modest. Future large-scale, multicenter prospective studies are warranted to confirm the diagnostic and prognostic value of IFITM proteins in a more generalizable population and to allow for more comprehensive adjustment of confounders.

## 5 Conclusion

Our study identifies serum IFITM1, IFITM2, and IFITM3 as novel and promising biomarkers for the diagnosis of CAD and AMI. The specific upregulation of IFITM1 in post-AMI heart failure suggests a potential distinct role in maladaptive remodeling. While these findings are compelling, they originate from a single-center cohort with inherent limitations such as potential referral bias. Therefore, future research should prioritize large-scale, prospective multicenter studies to validate the clinical utility of IFITMs, and further mechanistic investigations are warranted to elucidate their precise pathophysiological roles in atherosclerosis and post-infarction complications.

## Data Availability

The raw data supporting the conclusions of this article will be made available by the authors, without undue reservation.
