# Supplementary material for: Interferon-induced transmembrane proteins as biomarkers for assessing the diagnosis and severity of coronary artery disease and acute myocardial infarction
Source: Front Med (Lausanne). 2025 Nov 24;12:1645725. doi: 10.3389/fmed.2025.1645725 (PMC12682774; doi:10.3389/fmed.2025.1645725)
Supplement: Supplementary file 1 [file Supplementary_file_1.docx]

**Supplementary figures**


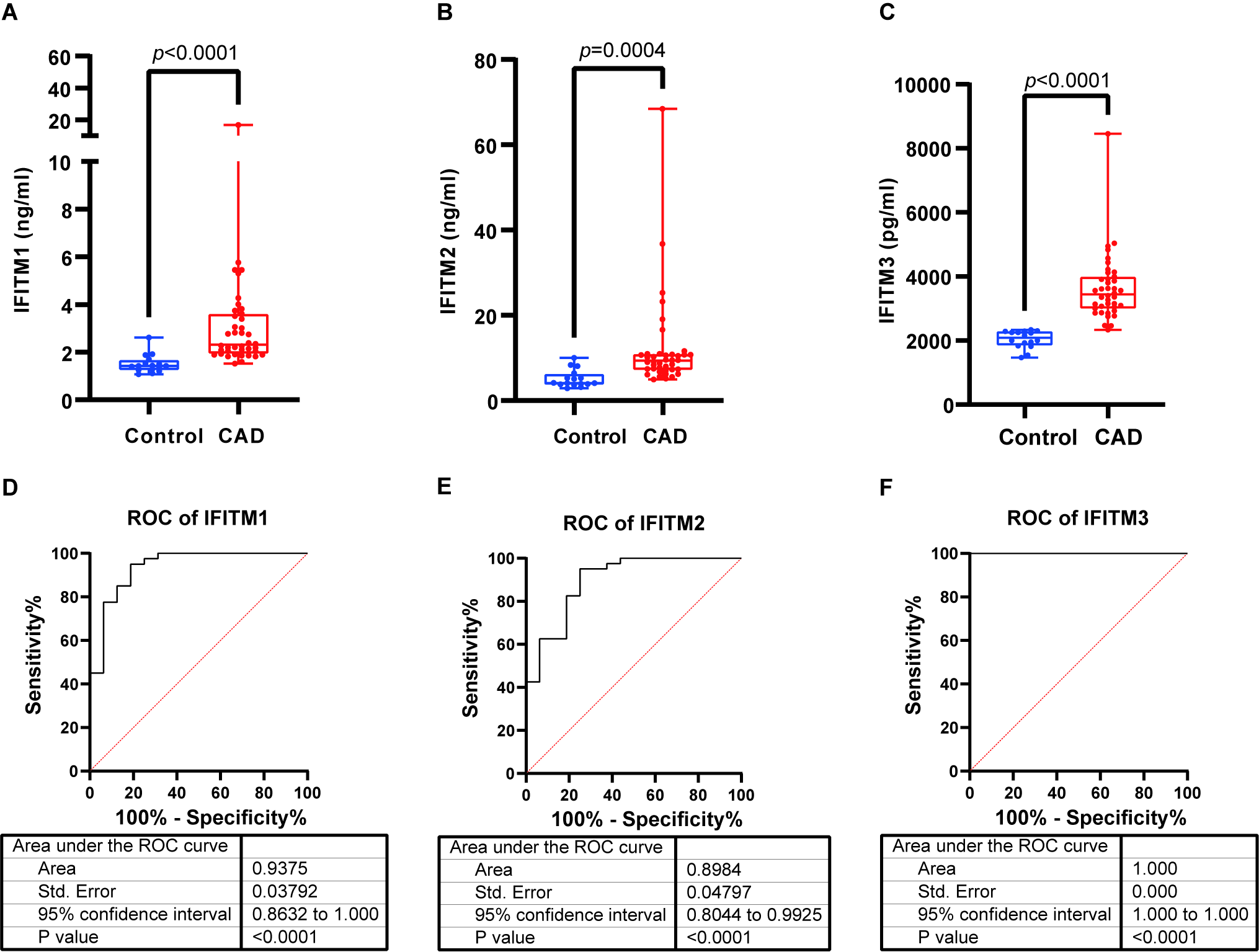


**Figure S1.** Serum levels and diagnostic performance of IFITM1/2/3 in control and CAD group of validation cohort. (A-C) Serum IFITM1/2/3 in control and CAD subjects; (D-F) ROC curves of IFITM1/2/3 for determination of CAD.Statistical significance was assessed using Wilcoxon rank-sum test.


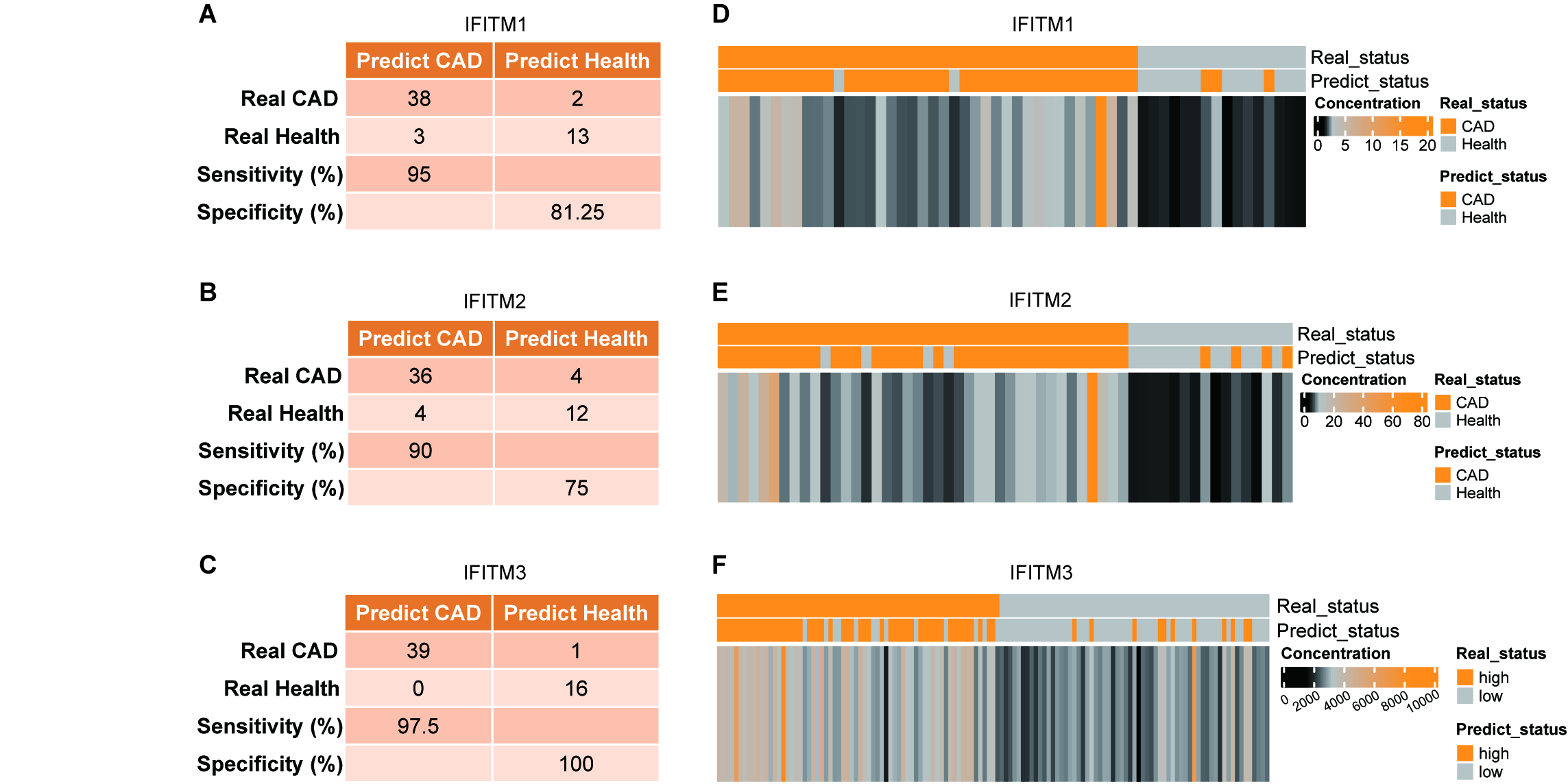


**Figure S2.** Diagnostic ability of IFITM1/2/3 in validation cohort. (A-C) Confusion tables of the binary results of IFITM1/2/3; (D-F) Supervised hierarchical clustering of IFITM1/2/3 between health control and CAD.


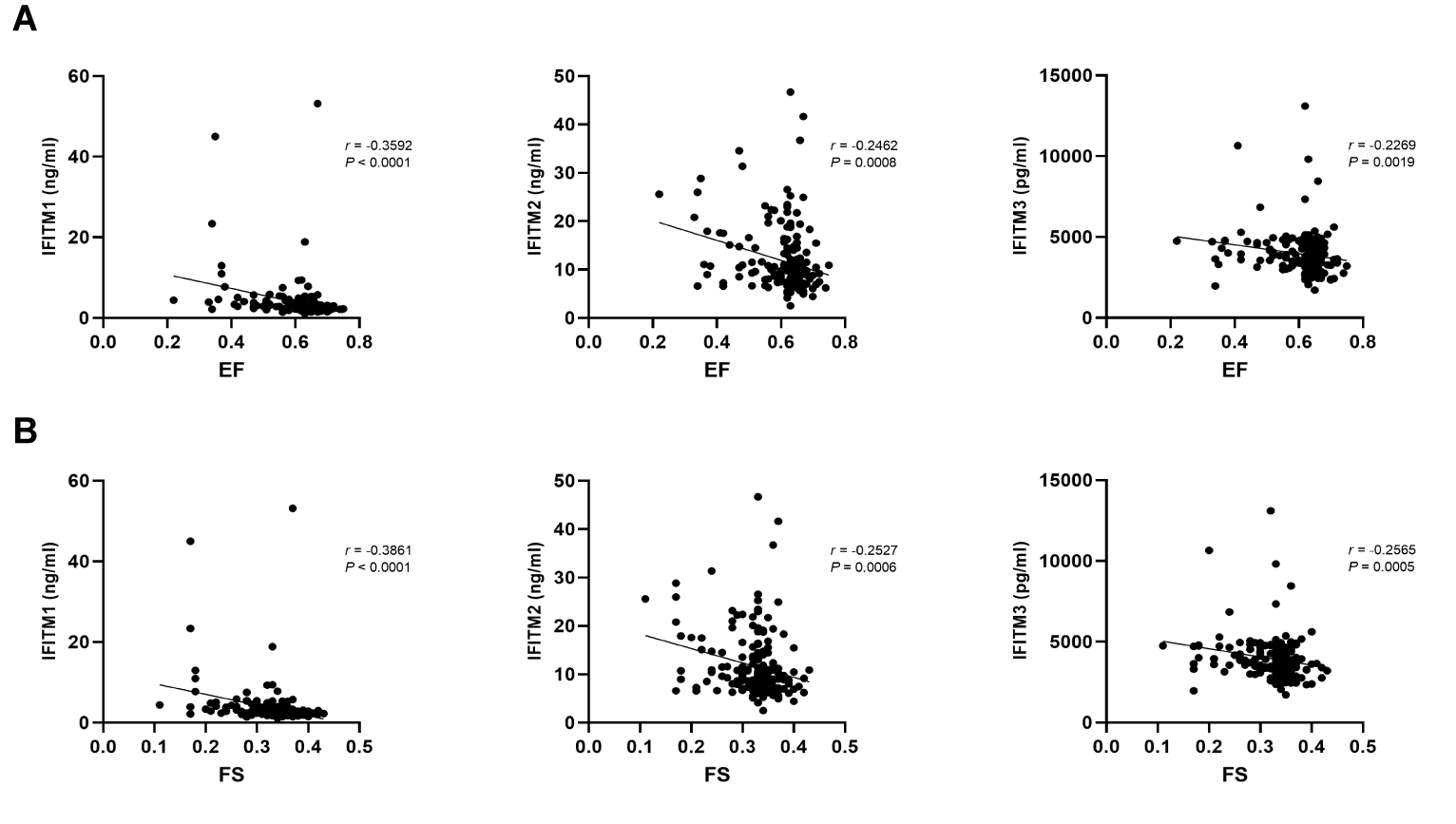


**Figure S3.** Correlation of serum IFITM1/2/3 with EF and FS.
