# Supplementary material for: Interferon-induced transmembrane proteins as biomarkers for assessing the diagnosis and severity of coronary artery disease and acute myocardial infarction
Source: Front Med (Lausanne). 2025 Nov 24;12:1645725. doi: 10.3389/fmed.2025.1645725 (PMC12682774; doi:10.3389/fmed.2025.1645725)
Supplement: Supplementary file 2 [file Table_1.docx]

Table S1 Demographic characteristics between AMI-nonHF and AMI-HF group

| **Variables** | **AMI-nonHF (n=30)** | **AMI-HF(n=22)** | **P value** |
| --- | --- | --- | --- |
| Gender |  |  | 1 |
| Female (n,%) | 7 (22.58%) | 4 (19.05%) |  |
| Male (n,%) | 24 (77.42%) | 17 (80.95%) |  |
| Age (year) | 66.00 (63.50-69.00) | 68.00 (65.00-70.00) | 0.197 |
| Diabetes (n,%) | 6 (22%) | 10 (63%) | 0.008 |
| Hypertension (n,%) | 23 (79%) | 17 (85%) | 0.7 |
| GensiniScore | 70.00 (35.00-80.00) | 64.00 (52.00-82.00) | 0.601 |
| White Blood Cells (× 10^9^/L) | 9.00 (7.30-10.40) | 8.90 (7.50-12.00) | 0.933 |
| Neutrophils (× 10^9^/L) | 7.03 (5.41-8.23) | 6.68 (5.17-10.00) | 0.874 |
| Lymphocytes (× 10^9^/L) | 1.29 (0.92-1.79) | 1.20 (0.87-1.71) | 0.73 |
| Monocytes (× 10^9^/L) | 0.49 (0.36-0.66) | 0.59 (0.41-0.66) | 0.288 |
| Red Blood Cells (× 10^10^/L) | 4.55 (4.24-4.87) | 4.41 (4.05-4.58) | 0.201 |
| Platelets (× 10^9^/L) | 191.00 (166.00-237.50) | 219.00 (191.00-268.00) | 0.138 |
| Troponin (ng/mL) | 4.15 (0.69-33.02) | 6.83 (1.45-30.69) | 0.568 |
| B Type Natriuretic Peptide (pg/mL) | 95.60 (46.80-264.75) | 685.50 (235.10-1175.90) | < 0.001 |
| C Reactive Protein (mg/L) | 8.19 (3.66-18.13) | 27.75 (17.29-66.02) | 0.057 |
| Creatinine (μmol/L) | 82.00 (72.55-93.85) | 88.60 (72.60-97.40) | 0.73 |
| Alanine Aminotransferase (U/L) | 27.00 (18.50-45.50) | 34.00 (24.00-59.25) | 0.263 |
| Aspartate Aminotransferase (U/L) | 39.00 (25.00-186.00) | 48.00 (32.00-81.00) | 0.867 |
| Creatine Kinase (U/L) | 253.00 (109.50-1336.50) | 217.00 (119.00-616.00) | 0.544 |
| Creatine Kinase MB Isoenzyme (U/L) | 33.80 (21.60-152.45) | 29.10 (18.80-62.40) | 0.376 |
| Triglycerides (mmol/L) | 1.69 (1.12-2.29) | 1.34 (1.04-1.85) | 0.168 |
| Total Cholesterol (mmol/L) | 4.43 (3.98-5.20) | 3.86 (3.42-5.04) | 0.076 |
| High-Density Lipoprotein (mmol/L) | 1.20 (1.06-1.44) | 1.23 (0.96-1.35) | 0.582 |
| Low-Density Lipoprotein (mmol/L) | 2.89 (2.55-3.26) | 2.64 (2.04-3.24) | 0.203 |
| Apolipoprotein A1 (g/L) | 1.05 (0.95-1.29) | 1.00 (0.83-1.12) | 0.07 |
| Apolipoprotein B (g/L) | 0.96 (0.78-1.12) | 0.81 (0.66-0.96) | 0.068 |
| Homocysteine (μmol/L) | 9.95 (8.47-11.87) | 11.36 (8.70-14.19) | 0.154 |
| IFITM1 (ng/mL) | 3.07 (2.80-3.65) | 4.64 (3.85-5.87) | 0.003 |
| IFITM2 (ng/mL) | 10.44 (8.66-16.51) | 15.12 (10.72-20.83) | 0.078 |
| IFITM3 (pg/mL) | 4445.05 (3583.18-4726.44) | 4717.05 (3982.11-4948.75) | 0.17 |

Data are presented as mean ± SD, median (IQR), or n (%) as appropriate; group comparisons were performed using Student's t-test, Wilcoxon rank-sum test, or Chi-square test based on data distribution and variable type.
